# Predator–Prey Dynamics Between Eurasian Sparrowhawk and Its Bird Prey During Spring Migration in the Forests at Hel Peninsula (N Poland) over 1982–2024

**DOI:** 10.3390/ani16040627

**Published:** 2026-02-16

**Authors:** Kamila Cymerman, Magdalena Remisiewicz

**Affiliations:** Bird Migration Research Station, Faculty of Biology, University of Gdańsk, Wita Stwosza 59, 80-308 Gdańsk, Poland; kamcym6@gmail.com

**Keywords:** predator–prey interaction, Eurasian Sparrowhawk, bird migration, spring phenology, climate change, Baltic region

## Abstract

Climate change in Europe can influence the predator–prey interactions, a scarcely studied topic in birds. We examined relationships between the spring passage timing of the Eurasian Sparrowhawk, a generalist peak predator, and its main bird prey species (Song Thrush, Eurasian Blackbird, Common Chaffinch, Great Tit, European Robin). All species were ringed daily (26 March–15 May) in 1982–2021 at Hel Peninsula (N Poland). In 2024, we collected pluckings to identify Sparrowhawks’ prey. Sparrowhawks’ daily migration dynamics and those for the Song Thrush, Robin and Chaffinch were correlated. Sparrowhawks’ median dates of passage showed no multi-year trend, but large variation, correlated with those of its prey and with temperatures. Adult females and males migrated through Hel early after warm February and March at wintering grounds. Young males migrated early during warm Aprils at Hel. Medians were correlated between adult males and Robins, adult females and Blackbirds, and young females and Song Thrush. This suggests that Sparrowhawks adjust their migration timing each spring to the availability of these three prey species, with some sex differences, as males are smaller than females. Identifying changes in predator–prey dynamics in response to climate change helps us understand its effect on forest ecosystems.

## Simple Summary

We investigated how the spring migration timing of the Eurasian Sparrowhawk is related to the timing of its five main bird prey species, considering climate change in Europe, which should affect both prey and the predator. Using bird ringing data (26 March–15 May, 1982–2021) at a stopover site Hel (N Poland), we found that adult Sparrowhawks migrated on average two to three weeks ahead of youngs. Young females, which can mate with older males, migrated 10 days ahead of young males. Adults occurred at Hel early following a warm February–March at wintering grounds in southern Europe. Migration timing of female Sparrowhawks (the larger sex) was related to that of large prey: Blackbird (adults) and Song Thrush (youngs). Adult males’ timing was related to Robins (small birds); young males showed no such relationships, but they migrated later, when all prey species were available. Robins and Song Thrush shifted spring passage by a few days over 1982–2021, but not Sparrowhawks. We suggest that Sparrowhawks (generalist predators) adjust migration timing each spring to some prey, but their phenology has not shifted, as they hunt various species. Understanding predator–prey dynamics in forests is important in the face of climate change.

## 1. Introduction

Bird migration phenology can be influenced by seasonal variation in food availability and weather conditions such as temperature, wind, and precipitation [1]. Furthermore, in recent decades, bird migration phenology has been adjusting to temperature changes due to global warming [2], resulting in shifts in breeding and migration periods [2,3,4,5,6,7,8,9,10]. Climate-related changes in spring migration timing have been described for many migratory bird species in Europe and North America as carry-over effects of changes in environmental conditions, such as availability of food resources or climatic conditions in different areas they visit at subsequent stages of their migratory life [8,11,12,13,14,15,16,17,18].

Climate change may also influence multispecies interactions [9,19,20,21], which are crucial for regulating and maintaining healthy ecosystems [9,22]. Changes in such interactions may vary across species and their relationships at different levels of the trophic chain, as organisms respond differently to changes in temperature or other environmental factors [9]. Predators are vital in regulating the population of their prey, making predator–prey interactions one of the most important in the trophic chain, at both the population and ecosystem level [9,23]. Climate change may influence the population dynamics of both predators and prey in the following ways: changes in range, population density, behaviour and phenology [9]. Multiannual data (1985–2005) from the Netherlands showed changes in the food chain at three levels: from caterpillars (an average 0.25 day delay compared to the budding of the plants they fed on) and passerines (an average 0.5 day delay in relation to the peak in caterpillar abundance) to predators, among which the Eurasian Sparrowhawk *Accipiter nisus* Linnaeus, 1758 (hereafter Sparrowhawk), showed the greatest mismatch between its breeding season and the hatching time of its passerine prey [9,24]. Raptor migration routes may have evolved in tandem with routes of their prey species [25,26]. The mismatch in the timing of predator and prey phenology, caused by climate warming, may have a greater impact on prey species, which shift spring migration and breeding earlier in response to changes in temperature and earlier availability of their plant and insect food, resulting in a temporal “escape” from the predator [24]. In North America, advances in spring migration timing, associated with climate change, have been observed for five out of the ten most abundant raptor species [8]. Furthermore, temperature changes in Europe influenced autumn migration dates of seven short-distance migrant raptor species, including the Sparrowhawk, which adjusted the timing of their autumn migration to temperatures during their breeding and non-breeding seasons during a 30-year study (1980–2010) in Western Europe [27]. In the Baltic region, long-term phenological shifts in the timing of spring migration since the 1960s until present were related to increased winter and spring temperatures at non-breeding grounds, for the Song Thrush *Turdus philomelos* C. L. Brehm, 1831 [28], European Robin *Erithacus rubecula* Linnaeus, 1758 [29] (hereafter Robin), Northern Wren *Troglodytes troglodytes* Linnaeus, 1758 [18], and Common Chaffinch *Fringilla coelebs* Linnaeus, 1758 [30] (hereafter Chaffinch). Recently, changes in spring migration timing have also been revealed in the Sparrowhawk [31,32]. With this in mind, we expect that raptors, including the Sparrowhawk, should adjust their spring migration dates according to the changes in migration timing of their prey species, which occur in response to variation in temperature.

Predators can be classified based on their prey selection. Specialists hunt a narrow range of species and are primarily responsible for regulating the population of their prey species. Generalists hunt a wider range of prey species, and a decline in the population of one prey species does not pose a threat to them, as they can prey on other species according to their abundance [9,33]. As generalist predators hunt a larger number of species, their population size should be more stable over time, and they ought to exhibit a higher rate of adaptability to climate change than specialists [9,34]. Despite higher population stability, generalists may also be affected by changes in the diversity or abundance of their prey, and the substitution of prey species can affect their interactions with other prey species or predators [9,35,36]. For example, Eleonora’s Falcon *Falco eleonorae* Géné, 1839, is a generalist bird of prey that, by a delayed breeding, relies on passerines and other small birds migrating in autumn through the Mediterranean basin or other areas to raise offspring [37,38,39]. In such raptors, shifts in autumn migration phenology of prey species can cause changes in behaviour and the timing of breeding. However, knowledge on the responses of generalist predators to changes in the phenology of their prey is limited [9].

We aimed to study interactions between Sparrowhawks and selected passerine prey species they hunt during stopover on spring migration along the Hel Peninsula (southern coast of the Baltic Sea), which concentrates the passage of both predatory and prey migrant birds (scientific names follow [40]). Considering that some passerines showed long-term trends and interannual variation in spring migration timing, we expected that Sparrowhawk’s timing would follow variation in their prey phenology, and we aimed to test this hypothesis. Thus, we set out to identify relationships between the timing of spring migration of this predator and its prey species within selected springs, and over the 40 years we studied, for Sparrowhawks as a whole, and for its age and sex groups. We also aimed to test whether increasing temperatures in Europe might be related to the advance of this species’ spring migration, as in its prey species. Thus, we aimed to identify the relationships between its annual variation in migration timing at Hel and temperature at Sparrowhawk’s wintering grounds, migration routes and at Hel Peninsula. To the best of our knowledge, these topics were not addressed for the Sparrowhawk before.

## 2. Materials and Methods

### 2.1. Study Site and Methods of Fieldwork

**Figure 1:**
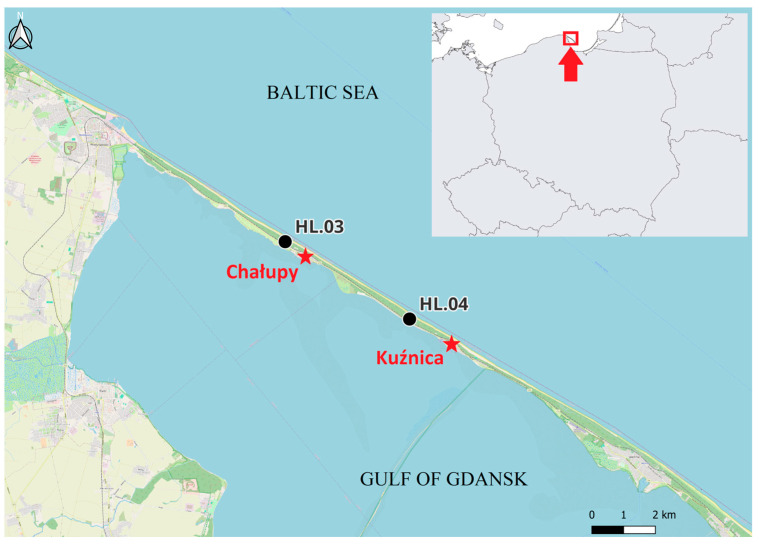
Location of the Hel bird ringing station, and of the villages between which the pluckings of Sparrowhawk were collected, at Hel Peninsula, N Poland. Black circles: HL.03—location of the Hel ringing station in 1982–1999 (54°45′47″ N, 18°30′00″ E), HL.04—location from 2000 until present (54°44′29″ N, 18°33′40″ E), red stars—villages near the bird ringing station, between which the pluckings were collected along a ca. 5 km forest path. The red arrow and square in the insert indicate the location of the study area on the Baltic coast of Poland.

The data used in this study were collected during the spring migration of birds (26 March–15 May) in 1982–2021, and in 2024, at the Hel bird ringing station, located on the Hel Peninsula (N Poland) (Figure 1), within the Operation Baltic Project [41]. Between 1982 and 2024, the station moved within 5 km along the Hel Peninsula, adjusting to the growth of forest, from location HL.03 near the village of Chałupy, where the station operated in 1982–1999, to location HL.04 near Kuźnica, where it has operated since spring 2000 to this day (Figure 1). The vicinity of the ringing station in both locations consisted of a pine forest with willow and rugose *Rosa rugosa* Thunb., 1784, shrubs in which the birds were captured in mist nets [42]. Passerines were caught in mist nets of 16–19 mm mesh and lengths of 7 m and 12 m, whereas most Sparrowhawks were caught in 12 m long nets with larger mesh (40–80 mm) [43]. The “raptor” mist nets at both catching locations were set up at similar, slightly open spaces in the coniferous forest, like the edge of forest clearings or across small paths. The “passerine” nets were open in places with denser vegetation, among bushes and tree canopies. However, as pine trees grew taller over the years the tree canopy has been gradually moving above the mist nets. This eventually enforced moving mist-netting to younger stands. These changes, as well as forest management activities, might have caused some changes in microhabitats between different springs, and influenced, to some extent, the numbers of caught birds, which we were not able to avoid. The number of mist nets of each type at the Hel station was stable throughout each spring season, but varied from 36 to 58 between years. We addressed that, and other potential sources, of the sampling bias, which might have affected the numbers of caught birds, by calculating migration parameters relative to the catching totals each spring. After extracting birds from the nets, they were ringed, their species, sex and age (if possible) were determined, and they were measured according to the Operation Baltic protocol [43]. Species, age and sex of each bird were identified by a qualified bird ringer according to plumage features using the identification guides [44,45].

To determine the Sparrowhawk diet during spring migration and select its bird prey species for further analysis, we collected pluckings and observed Sparrowhawk’s attacks on other birds along the Hel Peninsula, which is a stopover place for the raptor and its prey species. The Northern Goshawk *Accipiter gentilis*, Linnaeus, 1758, (hereafter Goshawk) and other birds of prey were seen at the study area on passage, but no attacks of the Goshawk, or any interactions between other raptors and the Sparrowhawk, were noticed. Pluckings and observations were collected in 2024 between 26 March and 12 May near the Hel ringing station (Figure 1) by KC and station’s volunteers, during regular net checks, in a pine sapling stand near the station where no mist nets were opened but which was an attractive place for bird predators, and along a ca. 5 km path in the forest between the villages of Chałupy and Kuźnica (Figure 1).

### 2.2. Study Species

**Figure 2:**
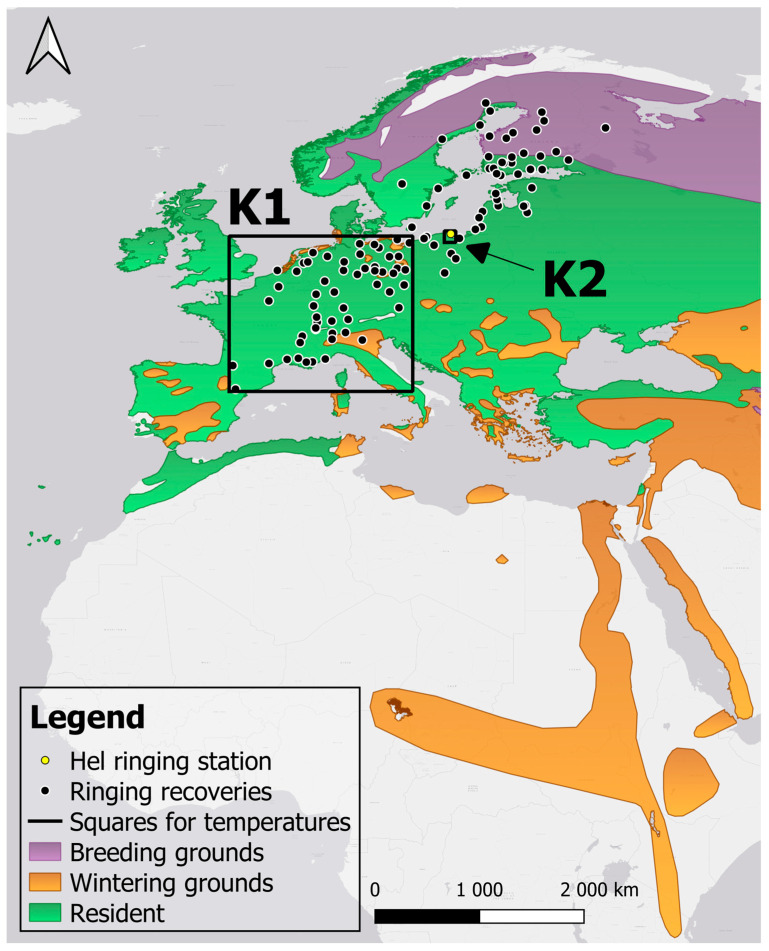
The geographical range of the Eurasian Sparrowhawk in Europe and Africa, and the location of the Hel ringing station, ringing recoveries of Sparrowhawks caught at Hel and two other Operation Baltic ringing stations on the Baltic coast of Poland, and areas for which we used mean temperatures of February–April. Square K1 (54.55–41.05 N, 0.69 W–15.26 E) includes wintering grounds and migration routes of the Sparrowhawk, according to ringing recoveries; square K2 (54.00–55.00 N, 18.00–19.00 E) includes the Hel station. Map of the species range after BirdLife International [51], modified.

The Eurasian Sparrowhawk is a medium-sized bird of prey, common across Europe and Asia, that primarily hunts passerine birds [46,47]. The main habitat of this species is woodlands with pine and spruce, where it builds nests, close to open areas, where it hunts. The breeding grounds of the Sparrowhawk extend across northern Europe, Russia and central Asia, and its non-breeding grounds span central and southern Europe, Africa, the Middle East, southern Asia, India, and south-western China [47] (Figure 2). The Sparrowhawk is the most commonly caught bird of prey during spring ringing at the Hel station. We focus on the populations of Sparrowhawk that migrate in spring to the north, from their wintering grounds in south-western Europe, through the southern coast of the Baltic, including the Hel station, towards their breeding grounds in Finland, Sweden, Norway and western Russia, as indicated by ringing recoveries (Figure 2) [48]. Sparrowhawks leave their wintering grounds in late February to mid-March, their peak passage through Europe occurs during March–April, and arrivals in their northern breeding grounds may extend until late May, and autumn migration begins in mid-August and lasts until early November [49]. Sparrowhawks can breed already at the end of their first year of life [49]. This species exhibits clear sexual dimorphism in plumage and size, with adult females being larger than males by up to 25%, and reaching the size of the Goshawk [49]. Male Sparrowhawks’ diet consists of prey of a weight of 40–120 g, and females can catch prey up to 500 g [49]. The diversity of species in the Sparrowhawk’s diet depends on the local availability of species [46]. In most European countries, the Sparrowhawk is a species of Least Concern (LC) status, with stable or increasing population numbers [50].

### 2.3. Criteria for Selecting Prey Species for Analysis

Most studies on Sparrowhawks’ diet focus on the breeding period, when birds form 77.2–97.4% of their prey items [35,46,52,53,54,55,56,57,58,59,60,61]. We selected the bird species analysed in this study as the Sparrowhawk prey based on these studies (Table 1 and Table 2), and on analysis of Sparrowhawk pluckings collected during its spring migration at the Hel Peninsula in 2024. All prey species we selected for analysis, which are briefly described below (Table 1), belong to the order Passeriformes.

### 2.4. Datasets and Methods of Their Collection

#### 2.4.1. Collection of Pluckings and Observations of Sparrowhawk Attacks

In 2024, from 26th March to 12th May, 48 pluckings of Sparrowhawks were collected around the Hel station. During this period, attacks on birds by the Sparrowhawk were also observed near mist nets by volunteers, or this predator was caught in the mist nets along with the prey. These observations, along with information on the prey species, the predator’s age and sex (if possible), and the location of the observation, were recorded at the ringing station. During the study period, 16 such attacks by Sparrowhawk were observed.

Pluckings are feathers remaining after birds of prey or owls pluck them out of their prey (also called pluckings) [64,65]. Pluckings by birds can be distinguished from those left by mammal predators by the tooth marks and damage they do to feathers; birds’ bills and claws leave only small holes in feather quills [64]. Pluckings by birds of prey can be distinguished between those left by the Goshawk and by the Sparrowhawk, as the latter species mainly plucks its prey, smaller than for the first species, on a log, stump, or other elevated location, among leaves in a tree, in an old nest [64] or near a tree in a dense forest [66]. The Sparrowhawk plucks its prey in one spot, pulling out feathers one by one, so they are concentrated in one place [66]. In contrast, when the Goshawk plucks feathers, it changes position and moves, scattering the feathers, and it plucks feathers more often in tufts than singly [66].

Male Sparrowhawks mostly feed on prey weighing 40–120 g, and female Sparrowhawks prey upon species up to even 500 g [49], so pluckings of larger and heavier species found in the study area, like Long-tailed Duck *Clangula hyemalis,* Linnaeus, 1758, were not considered in the analysis. Because other birds of prey, such as the Goshawk, occurred in the area, as well as various species of owls and mammals, the entire appearance of the plucking site and feather arrangement were examined by KC, either personally or based on photos provided by volunteers. The species of the predator and prey were identified by KC’s own observations and knowledge, using the Featherbase website [67].

#### 2.4.2. Materials Collected During Bird Ringing

For all six study species, we extracted from the Operation Baltic database the dates of their first capture at the Hel station during the springs of 1982–2021; for the Sparrowhawk, we also extracted the age and sex of the analysed birds (Table 3). For the Sparrowhawk, for each sex, we merged birds aged in the field as juveniles and immatures, which were all in spring in their second calendar year of life, into the age group of “immatures” (young birds). Sparrowhawks that were aged as adults (older than two years), or occasionally as birds in their third year of life, were also merged as “adults”, also separately for each sex. For the five passerine species, we analysed immatures and adults combined (Table 3).

#### 2.4.3. Temperatures Along Sparrowhawk Spring Migration Routes

To determine if the timing of Sparrowhawks’ passage at Hel was related to temperatures at their wintering grounds and migration routes, we used temperatures in February–April in regions K1 and K2, defined by squares of coordinates (Figure 2). We used temperatures from February and March in the square K1, including the area of south-western Europe where Sparrowhawks ringed on the Polish coast of the Baltic Sea were recovered (Figure 2), as a proxy for temperatures at their departures from wintering grounds and along spring migration routes. We used the temperature in April within a one-degree grid square K2 that includes the Hel station, to reflect conditions on their arrival at that location. We downloaded daily mean temperatures within the selected ranges of coordinates (Figure 2) from the ERA5 dataset using the Climate Explorer facility of the World Meteorological Organisation [68]. We then averaged these daily temperatures for the selected months. Temperatures from the same months in areas K1 and K2 were strongly correlated (Table A1), so we avoided using them in one model.

#### 2.4.4. Statistical Analysis

Based on the daily ringing data for each species, we summed the number of birds captured each day of the spring season (26 March–15 May) in subsequent years from 1982 to 2021. For the Sparrowhawk, we also summed the daily numbers of individuals ringed within four age and sex groups: adult males, adult females, immature males, and immature females. For each species, using these daily totals, we calculated the percentage of birds captured on each day of the season relative to the total number of birds caught that spring, to draw the daily migration dynamics in each season. These percentage daily dynamics allowed for comparison of migration patterns between seasons in which small and large numbers of a species were caught. For the Sparrowhawk, we also calculated and drew daily migration dynamics for each age/sex group. Then, to derive the many-year average daily migration dynamics for each age/sex group of Sparrowhawk, we calculated the average proportion of birds captured during 1982–2021 each day of the spring season. We obtained the multi-year average spring migration dynamics for each studied species in the same way.

To determine if Sparrowhawk migration coincided with the migration period of prey, for each prey species, and for each age/sex group of Sparrowhawk, we calculated the date (the day number in the year, 1 January = day 1) on which 25% (q25) of individuals were ringed during each spring in 1982–2021. We repeated the same procedure for 50% (q50 = median) and 75% (q75) of the ringed birds. We excluded from these calculations springs when fewer than 10 birds of a species or group were caught. To present the overall median date of passage for each species or sex/age group we calculated the median of all the yearly median dates in 1982–2021. Analogously, we calculated the overall dates of q25 and q75 as the median of the respective dates across all years. This way each year had the same weight in calculating the multi-year quartiles, irrespective of the number of individuals caught each spring.

To compare the dates of passage of subsequent quartiles (q25, q50, q75) of the ringed birds between the Sparrowhawk and its prey species, we run three multiple regression models, one for each quartile. In each model, the dates of the selected quartile over 1982–2021 for the Sparrowhawk (all individuals jointly) were the response variable, and analogous dates for the five prey species were explanatory variables. Then, we repeated this modelling procedure for each age/sex group of the Sparrowhawk. We applied the “all subsets regression” procedure and selected the best model by the Akaike Information Criteria (AIC) using Statistica 13.3 [69]. To relate the timing of passage of Sparrowhawks to temperatures at their wintering grounds and migration routes, for each sex and age group we run a multiple regression model, with the median dates (q50) of their spring passage in 1982–2021 as the response variable and the monthly temperatures of February–March in the square K1, and of April in the square K2, as explanatory variables. Analogously to the previous multiple regression models, we selected the best model using “all subsets regression” and AIC. These methods are analogous to those used in other studies using the long-term data from the Operation Baltic Project [18,70,71].

To better understand the relationship between predator and prey migration in each season, we compared the daily migration dynamic of selected prey species and of Sparrowhawks (all groups combined) during seven springs in which more than 100 Sparrowhawks were ringed (Table A2), using Kendall’s Tau correlation coefficient. In this way, for each prey species, we obtained seven correlation coefficients with Sparrowhawk dynamics, one for each selected spring. Thus, we applied the Bonferroni correction for multiple comparisons and used the adjusted level of significance *p* < 0.00714 to interpret these results. All statistical calculations were conducted in Statistica 13.3 [69], and maps were made in QGIS 3.28.5 [72].

## 3. Results

### 3.1. Prey Species in Pluckings and Observations of Attacks in Spring 2024

**Figure 3:**
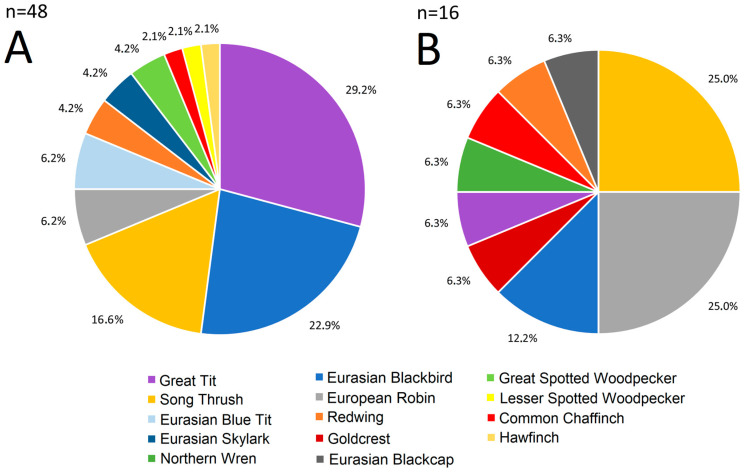
The diet of the Eurasian Sparrowhawk during spring migration through the Hel Peninsula between 26 March and 12 May 2024. (**A**) Proportions of species among pluckings identified as the Sparrowhawk prey, collected near the Hel bird ringing station. (**B**) Proportions of prey species among observations of attacks or dead birds in nets identified as the Sparrowhawk victims collected near the Hel station.

Among the pluckings collected in spring 2024, Great Tit dominated, followed by the Eurasian Blackbird, Linnaeus, 1758 (hereafter: Blackbird) and Song Thrush (Figure 3A, Table A3). Among the victims of observed Sparrowhawk attacks, Song Thrush and Robin predominated (Figure 3B, Table A4). These results (Figure 3) supported our initial choice based on the literature (Table 1). Thus, we selected five passerine species (Great Tit, Blackbird, Song Thrush, Robin, Chaffinch) for further analyses, as the main Sparrowhawk’s bird prey species on spring passage.

### 3.2. Spring Migration Timing of Sparrowhawks by Age and Sex

**Figure 4:**
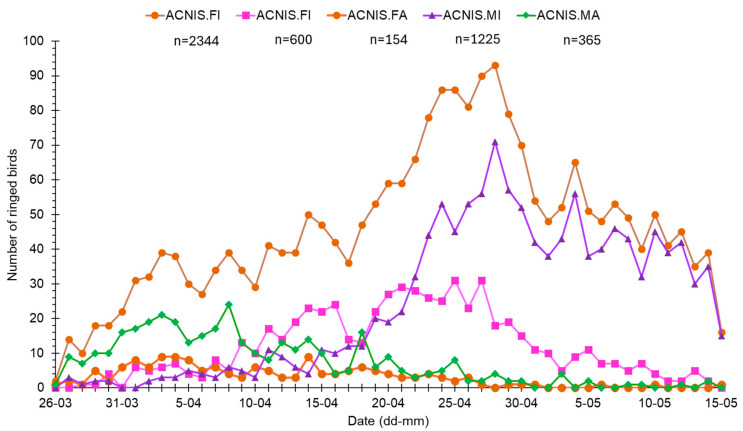
Numbers of Eurasian Sparrowhawks caught at Hel station (N Poland) during spring migration (26 March–15 May) in 1982–2021. ACNIS.FI = immature females, ACNIS.FA = adult females, ACNIS.MI = immature males, ACNIS.MA = adult males.

**Figure 5:**
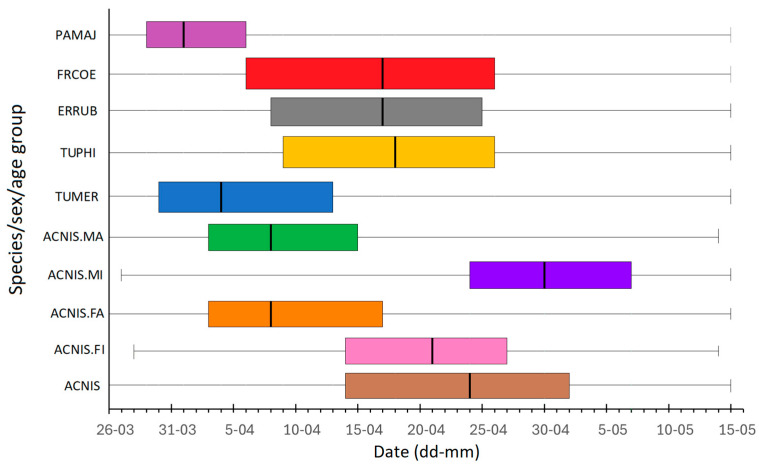
The timing of spring migration of the Eurasian Sparrowhawk, by age and sex, and for all groups combined, and of its potential prey species, based on spring (26 March–15 May) data from the birds ringing station Hel (N Poland) in 1982–2021. ACNIS = Sparrowhawk *Accipiter nisus* (all groups combined), ACNIS.FI = immature female Sparrowhawk, ACNIS.FA = adult female Sparrowhawk, ACNIS.MI = immature male Sparrowhawk, ACNIS.MA = adult male Sparrowhawk, TUMER = Eurasian Blackbird *Turdus merula*, TUPHI = Song Thrush *Turdus philomelos*, ERRUB = European Robin *Erithacus rubecula*, FRCOE = Common Chaffinch *Fringilla coelebs*, PAMAJ = Great Tit *Parus major*. Vertical line in the box = median (q50) of migration, box = q25–q75 of passage, whiskers = minimum and maximum migration dates.

The first Sparrowhawks in spring at Hel station were caught on 26 March, and their passage lasted until mid-May (Figure 4 and Figure 5). The timing of spring migration differed between age and sex groups of Sparrowhawks (Figure 4 and Figure 5). Adult males were the first caught Sparrowhawks, shortly followed by adult females, ahead of the first immatures (Figure 4 and Figure 5). The median dates of passage of adult males and females were similar (Figure 5). Most adult males migrated earlier than immature males (Figure 4), on average by 22 days (Figure 5). Adult females migrated on average 13 days earlier than immature females (Figure 5). Immature males migrated the latest and were caught in the largest numbers among all the sex/age groups of Sparrowhawks (Figure 4 and Figure 5).

**Figure 6:**
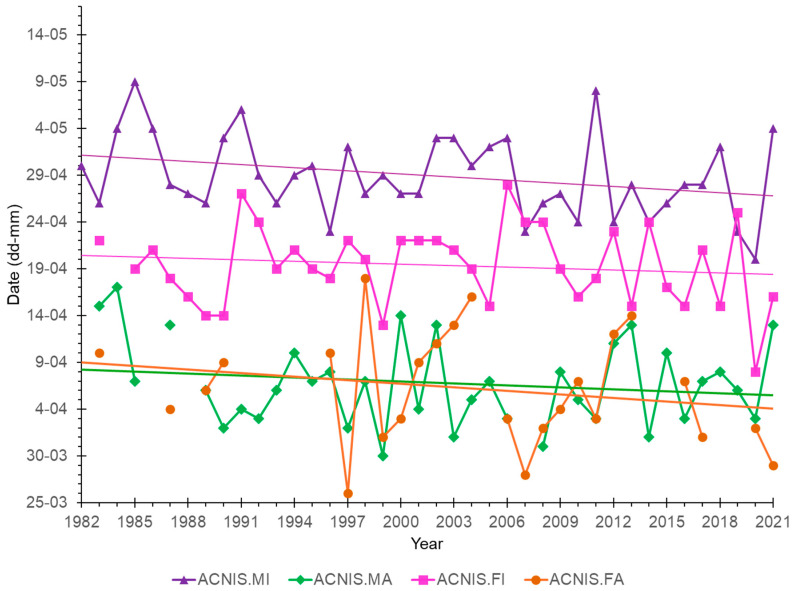
Multi-year trends in median dates of passage for the sex and age groups of Sparrowhawks caught during spring migration (26 March–15 May) at the Hel ringing station in 1982–2021. ACNIS.MI = immature males, ACNIS.MA = adult males, ACNIS.FI = immature females, ACNIS.FA = adult females, regression lines for each sex/age category in corresponding colours. None of the regression lines was significant. The details of the regression are presented in Table A5.

The overall migration timing of the Sparrowhawk showed no trend in the first (q25) and second (q50) quartiles, but the third (q75) quartile had a significant trend to earlier passage over 1982–2021 (β = −0.11, R^2^ = 0.11, *p* < 0.05). In all four sex/age groups of Sparrowhawks, the median dates of passage (q50) showed no significant trends over 1982–2021 (Figure 6, Table A5), but had large interannual variation, and were similar for adult males and females (Figure 6). For none of the prey species, the median dates (q50) of spring passage showed any significant trend over 1982–2021 (Table A6).

### 3.3. Relationships Between Spring Migration Timing of the Predator and Prey Species

#### 3.3.1. Relationships in Migration Timing over 1982–2021

The first and last Sparrowhawks were captured at the beginning and at the end of the spring bird ringing season at Hel station, similar to their prey species (Figure 5). The interquartile range q25–q75 when 50% of Sparrowhawks (all birds jointly) passed through Hel in 1982–2021 overlapped with analogous ranges for the Song Thrush, Robin and Chaffinch (Figure 5). For adult males and females, the passage of 50% individuals (q25–q75) also overlapped with that of the Great Tits and Blackbirds, which are early migrants at Hel (Figure 5). The dates for the first (q25) and the third (q75) quartiles of all Sparrowhawks’ passage in subsequent years of the period 1982–2021 were related to the corresponding dates for Robins, and the median date (q50) was related to that of the Song Thrush, according to the best multiple regression models (Table 4, Table A7, Table A8 and Table A9). For adult male Sparrowhawks, the median date (q50) during 1982–2021 was positively related to the median dates for Robin, according to the best model (Table 5), which indicated that the predator migrated early if this prey species migrated early and late when Robins migrated late. The median date for this group was also related negatively, though not significantly, to those for the Song Thrush (Table 5). For adult females, the median date (q50) of passage was positively related to that of the Blackbird. The median date for immature females was positively, though not significantly, related to that of the Song Thrush, and for immature males, the median was negatively associated with that for the Great Tit (Table 5, Table A10, Table A11, Table A12 and Table A13).

#### 3.3.2. Correlations of the Daily Migration Dynamics During Selected Springs

**Figure 7:**
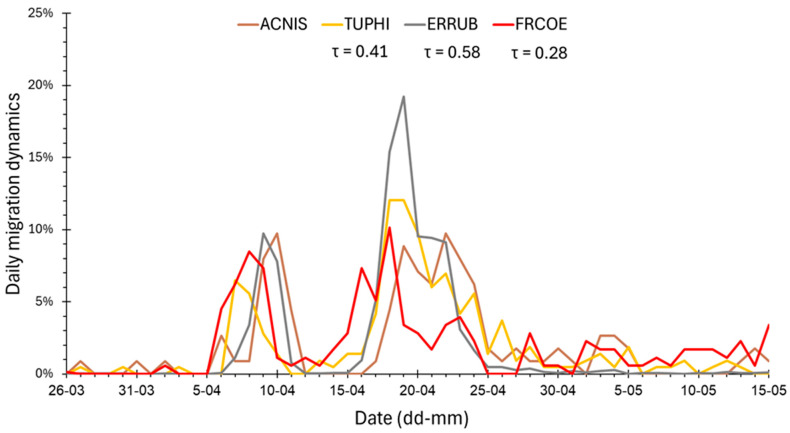
The daily dynamics of spring migration in 1996 at Hel of the Sparrowhawk (all groups jointly) and of the prey species, for which the dynamics were positively correlated. AC.NIS = Sparrowhawk *Accipiter nisus*, TUPHI = Song Thrush *Turdus philomelos*, ERRUB = European Robin *Erithacus rubecula*, FRCOE = Common Chaffinch *Fringilla coelebs*, τ = Tau Kendall correlation coefficients between the dynamics of the abbreviated species and the Sparrowhawk.

The daily migration dynamics of the Sparrowhawk were positively correlated with those for the Song Thrush and Robin in 1989, 1996, 1999, and 2000, and for the Song Thrush only in 2002 (Table 6, Figure 7). The correlation with Robin migration was the strongest in 1996 (Table 6), likely due to two peak periods (6–12 April and 15–23 April) of passage of both species (Figure 7). In 1996, the Sparrowhawk migration dynamics was also positively correlated with that of the Chaffinch (Table 6). In 1998, it was significantly and negatively, but weakly, correlated with the dynamics of the Blackbird, Chaffinch and Great Tit. In 2000, the Sparrowhawk daily migration dynamics was negatively correlated with that of the Blackbird, and in 2002 with the Great Tit only (Table 6).

### 3.4. Relationship Between Winter and Spring Temperatures and Spring Migration Timing of Sparrowhawk at Hel

For the immature male Sparrowhawk, the median dates (q50) of passage at Hel were early with high temperatures in April around the station (area K2 in Figure 1), according to the best model (Table 7). For adult males, the medians of passage at Hel were early with high March temperatures on their wintering grounds and migration routes (area K1), and vice versa. For adult females, the medians were early with warm February on wintering grounds and migration routes (area K1) (Table 7). For immature females, we found no analogous relationship.

## 4. Discussion

### 4.1. Sparrowhawk’s Diet During Spring Migration Through Hel Peninsula

Pluckings were collected in spring 2024 to help the choice of bird prey species for further analyses, which was initially based on the literature (Table 1). Pluckings’ collection and observations of Sparrowhawk three years later than the period of migration phenology analyses (1981–2021) might not have been representative for that period. However, the plucking collections were representative of all forest habitats in the studied part of the Hel Peninsula, as they were collected within the narrow forest belt along the Hel Peninsula between the past and the current location of mist-netting (Figure 1). We used results from pluckings collection and observations only to support the choice of prey species, which was initially based on the literature, which included the period 1981–2021. The prey species identified in pluckings collected at the spring stopover site around the Hel station corresponded well with bird species hunted by Sparrowhawks across Europe during the breeding season, including the years we studied [35,46,52,53,54,55,56,57,58,59,60,61], and all are common forest species, which supported our selection of the five main bird prey species. However, we cannot exclude the possibility that the Sparrowhawk diet on passage has somewhat changed over the 40 years we analysed, which we bear in mind while interpreting these results.

The number and diversity of collected pluckings may have been influenced by weather and the difficulty in finding feathers from smaller bird species. Due to the small size of feathers, such as those of the Great Tit and Eurasian Blue Tit, and their faint colours, it is possible that not all pluckings of small birds were found in the study area. Blackbird and Song Thrush feathers are larger and darker (black, brown) than those of the two species of tits, so they stand out more against the dense green undergrowth in the forest where the material was collected. The dominant species among plucking collected during 26 March–12 May in 2024 were Great Tits and Blackbirds, for which the main peaks of passage that spring occurred at the turn of March and April, when adult Sparrowhawks migrated through Hel (Operation Baltic unpubl. data). This suggests that Sparrowhawks passing through Hel later that spring probably hunted the late individuals of these prey species, which are usually immature, old or sick birds [1,31]. The presence of pluckings of other species, such as the Eurasian Blue Tit, Redwing, and Eurasian Skylark, besides the five main prey species, indicates that Sparrowhawk, as a generalist predator, hunts a wide variety of prey during spring migration through Hel (Figure 3). We did not include these other species in the analysis because they were absent or occurred in small numbers in the Sparrowhawk’s breeding diet, and were a few among birds ringed at Hel, except for the Blue Tit [35,46,52,53,54,55,56,57,58,59,60,61]. Many factors might have influenced the plucking collection, which was limited to one season, so these results should be treated cautiously and only qualitatively, as an indication of the preferred prey species of the Sparrowhawk during spring migration at Hel.

### 4.2. Migration Timing of the Sparrowhawk over 1982–2021

#### 4.2.1. Sex- and Age-Differential Timing of Spring Passage

Among all the analysed sex/age groups, adult males arrived at the Hel as the first Sparrowhawks, slightly before the first adult females (Figure 4). In this species, the male chooses the breeding site, even if both sexes participate in building the nest [64], which would explain this sequence of passage. The earlier male arrival on breeding grounds (protandry) has been observed in many birds, especially in monogamous species, such as the Sparrowhawk [73]. In such species, males are under greater selection pressure to arrive early on breeding grounds than females, to secure the best breeding territories [74], which might explain the occurrence of males as first Sparrowhawks at Hel. However, the median dates of spring passage, as well as the numbers of ringed adults of both sexes during 1982–2021, were similar (Figure 4, Figure 5), in line with females’ participation in nest building [64].

Adults migrated through Hel on average ahead of immatures, similarly to Helgoland (Germany), where adults migrated in the first half of April, and young migrated only in the second half of April [49]. This sequence of passage might be the effect of greater experience of adult Sparrowhawks, which enables them to arrive at breeding grounds earlier than immatures to establish breeding and hunting ranges abundant in prey and nesting material, or arrive at their established sites to defend them against immatures and to build a nest on a new tree each year [64]. Sparrowhawks in spring at the end of their first year of life are sexually mature and can attempt breeding, mostly with a mate of the same age; however, fewer young pairs have breeding success than their older counterparts [49]. Spring passage of adults ahead of the young was also observed in the Lesser Spotted Eagle *Clanga pomarina* Brehm, 1831, and Tawny Eagle *Aquila rapax Temminck, 1828*, in which immatures arrive at the breeding grounds six to ten weeks after adults [75], and in various eagle species observed during spring passage in Israel [76]. Having less experience, immatures migrate later in spring and, as a result, choose poorer territories than adults, but still try to reproduce. The difference in migration timing between adults and immatures also probably reduces their competition for food at stopovers, especially in early spring.

Among immatures, the median date of migration was on average 9 days earlier for females than for males (Figure 5), in contrast to adults. We found no literature record of such a pattern in immature Sparrowhawks. One explanation for that discrepancy in timing might be different food preferences of the sexes. The main prey of female Sparrowhawks are birds from families Turdidae and Sturnidae, whereas male Sparrowhawks hunt smaller birds from families Fringillidae and Paridae [49]. Our results showed that the median dates of spring passage of young female Sparrowhawks were related (Table 5), with those of the Song Thrush, and their main periods of passage (q25–q75) overlapped (Figure 4). The timing of spring passage of young males overlapped with the second half of the passage of all prey species (Figure 4), and males tend to hunt smaller prey, which is abundant throughout spring [49]. Risch and Brinkhof [77] showed that among 102 breeding pairs with known age for both sexes, studied in 1992–1996 in Northern Germany, 80 pairs were of the same age category (64 adult and 16 first-year pairs), but among the remaining 22 pairs, 20 were “adult male and first-year female” pairs [77]. Furthermore, females of any age class paired with adult males began egg laying earlier than females that mated with first-year males. During the reproductive period, the male provides food for the female and their offspring [46,49,77]. Immature males, which have less experience and probably poorer hunting grounds, provide less or lower-quality food than adults, extending the time required to raise chicks. Thus, immature females likely migrate earlier in spring than immature males to pair with an experienced male and increase breeding success.

We found a trend for earlier migration over 1982–2021 only for the third quartile (q75) of Sparrowhawk passage, which likely represents the shift in migration timing for immature males that dominate the last phase of the Sparrowhawk passage at Hel (Figure 4). In our study, no long-term trends in the median dates of passage for any sex and age group of Sparrowhawks were significant; however, all groups showed a tendency for earlier median dates of spring passage through Hel over 1982–2021, corresponding with the results from Hanko Bird Observatory in Finland (59°49′ N, 22°54′ E) [31]. Lehikoinen and co-authors [31] showed that the beginning of migration (the date of 5% of passage), which includes mostly adult Sparrowhawks, through Finland has advanced by 11 days over 1979–2007, while the dates of 50% and 95% migrating individuals did not show any analogous shift [31]. Thus, both in our study and in that at Hanko [31], some multi-year shifts to earlier spring passage occurred in Sparrowhawks, although for different phases of passage. The source of this discrepancy might be differences in the periods of these two studies, as the selection of years affects the resulting multi-year trend in migration phenology [28]. Additionally, the studies differed in the method used, as data from Hanko came from daily counts of migrating Sparrowhawks [31], and we used data from ringing. In the study on Sparrowhawks’ spring migration at Rybachy station at the Courish Spit on the Baltic Sea, about 150 km northeast of Hel, Sokolov and co-authors [78] showed that the spring passage of this raptor significantly advanced during 1958–1984, with the median passage date being 10 days earlier. However, no analogous trend occurred during 1985–2011, which is in line with our results from Hel and those from Hanko [31].

#### 4.2.2. Effect of Temperatures at Wintering Grounds and Migration Routes on Sparrowhawks’ Spring Migration at Hel

The Sparrowhawk is a short- or medium-distance migrant [46], and such migrants respond to climate change quicker than long-distance migrants, which overwinter far from their breeding grounds [79,80]. The influence of temperature on migration timing can vary between age and sex groups within one species [81]. In our study, the migration timing of each sex/age group of Sparrowhawks was related to temperature in different months and areas, but in the same way: early median dates (q50) of spring passage at Hel were related to high temperatures at the non-breeding grounds, and vice versa (Table 7). For adult females, the medians of passage at Hel were related to temperatures on wintering grounds and migration routes (K1 square at Figure 1) in February, and for the males in March, when Sparrowhawks begin their migration [82], as most adult Sparrowhawks were caught at Hel in the first half of April (Figure 5). These results suggest that warm end of winter at the non-breeding grounds promotes early migration of adults, probably because of lower energy expenditure on thermoregulation and increased hunting efficiency as their prey species also begin spring migration early after warm winters [5,6,7,28,29,30]. Warm winters in those areas might benefit females’ hunting and thus their faster accumulation of reserves required for migration, which should promote earlier departure from the wintering quarters and fast spring passage with few stopovers. As Sparrowhawk is a short- to medium-distance migrant, weather at wintering grounds before departure corresponds with conditions on route and at breeding grounds [74,83]. Hence, a warm March at wintering grounds might be an indication for adult male Sparrowhawks that spring is early and warm along the migration route, and likely also at the breeding grounds, which urges them to depart early and migrate as fast as possible, to occupy the best breeding territory.

In contrast, the median for immature males at Hel was early when April was warm near the station (K2 square), and they were the only sex/age group that responded to local temperatures, likely because they migrate through Hel the latest, between mid-April and mid-May (Figure 4 and Figure 5). This corresponds with the results from the Courish Spit, where local April temperatures and January–March NAO index were related to the early arrival of Sparrowhawks during warm springs [78]. On the other hand, Finnish researchers did not find any significant correlation between Sparrowhawks’ arrival dates and local temperatures in April, and suggested the influence of weather conditions at departure and migration routes [31], a relationship we showed for adult male and female Sparrowhawks at Hel.

### 4.3. Migration Timing of the Predator and of the Prey Species

The dates of q25 and q75 of Sparrowhawks passage (all individuals jointly) and the median (q50) date for adult males during 1982–2021 were positively related to analogous dates for the Robin (Table 4 and Table 5), and in three out of seven springs the daily migration dynamics of these species were correlated (Table 6), which point at Robin as an important prey for Sparrowhawk. Robins migrating through Hel ringing station advanced all phases of their spring passage (5%, 50%, 95%) over 1970–2018 [29], similarly to the situation at Helgoland station (North Sea, Germany, 1960–2000) [14] and on Christiansø island (Baltic Sea, Denmark, 1979–1997) [84]. However, we found no significant trend in the median dates of passage of Robin at Hel over 1982–2021, similarly to that of the Sparrowhawk, but both species showed strong year-to-year variation in spring phenology. Like the Sparrowhawk, Robin is a medium-distance migrant [29], and populations of these species that migrate through the southern Baltic coast share breeding grounds in Fennoscandia, the Baltic countries, and western Russia, and wintering grounds in the Iberian Peninsula and the Apennine Peninsula (Figure 2) [42]. The relationships between migration timing of Sparrowhawks and Robins in subsequent springs suggest that this predator adjusts its spring phenology to the interannual changes in phenology of that prey species.

The median (q50) dates of spring migration at Hel for Sparrowhawks (all birds) during 1982–2021 were related to analogous dates for Song Thrush, and in four out of seven chosen seasons, the daily migration dynamics were positively correlated between these species (Table 4 and Table 6). April is the main month of Song Thrush migration through the Baltic region [28,85], including Hel (Figure 5), similarly to most Sparrowhawks, which can then use the abundance of Song Thrush as prey. Warm February at the wintering grounds and warm April along migration routes were related to early spring passage of the Song Thrush at Hel [28,74], as in the Sparrowhawk. This similarity suggests that the Sparrowhawk might adjust its migration timing each spring to that of the Song Thrush, which is an attractive prey.

The passage of 50% of adult female Sparrowhawks (range q25–q75) overlapped with the main period of Blackbird passage through Hel, and the median dates of passage for adult females during 1982–2021 were related to those of Blackbirds (Table 5), which suggests females’ preference for this prey species. Female Sparrowhawks, being up to 25% larger than males, hunt larger prey, including birds of the Turdidae family [49], which corresponds well with our results.

The median dates of immature male Sparrowhawk migration at Hel negatively correlated with those for the Great Tit (Table 5), and daily migration dynamics of both species were negatively correlated in 1998 and 2002 (Table 6). Most Great Tits migrate through the Baltic region early in spring [86], as confirmed by our results, which showed that 50% of this species’ passage at Hel was cumulated within 9 days at the turn of March and April (Figure 5). Such timing of Great Tit passage differed the most from that of immature male Sparrowhawks, for which the first quarter of individuals (q25) passes through Hel on average at the end of April, when most Great Tits are already gone (Figure 5). Thus, these negative correlations likely result from a very different time of migration of these species, rather than from young Sparrowhawks “avoiding” Great Tits. Among the collected pluckings, Great Tits dominated (Figure 3), which might have been caused by Sparrowhawks preying upon individuals that migrate late, which are likely young or sick individuals [1,31] that are easy prey for the raptor.

The daily migration dynamics of Chaffinch, as the only one of the five prey we studied was correlated positively (in 1996) and negatively (in 1998) with the dynamics of the Sparrowhawk’s migration. The Chaffinch migration at Hel, like that of the Sparrowhawk, shows large year-to-year variation in timing and numbers related to temperatures at its non-breeding grounds [30], which may explain the different signs of the correlation in those years. In spring 1998, most Chaffinches migrated early, ahead of most Sparrowhawks, hence the negative correlation of their migration dynamics, similar to the Great Tits. But in 1996, Chaffinches were more numerous at Hel than in 1998 (Table A2), and their main migration occurred in April, with peaks on similar days to those of Sparrowhawks, which explains the positive correlation and suggests that Sparrowhawks used the abundance of Chaffinches as their prey at that time.

## 5. Conclusions

Our results showed that the year-to-year changes in spring migration timing of Sparrowhawks were related to those of their prey species, with some differences between age and sex groups of the predator, likely reflecting their food preferences linked to their sexual dimorphism in size. Our results suggest that Sparrowhawks may adjust their migration timing each spring to match the current availability of their prey species, which in turn is influenced by the temperature and conditions at their non-breeding grounds. We found no long-term shifts in Sparrowhawk’s spring migration phenology over the three decades, in spite of such trends occurring for its prey species, probably because, as a generalist predator, it is rather flexible in its prey choice. However, with further progress of the climate change, and similar shifts in spring phenology in several prey species, we can expect eventually earlier spring passage of the Sparrowhawk in northern Europe. Sparrowhawks are among the peak bird predators, which regulate the population size of their prey species in forests. Thus, identifying changes in predator–prey dynamics of that species in the face of climate change is key to understanding the effect of such changes on forest ecosystems. Long-term monitoring at bird ringing stations located at bird stopover sites provides valuable data that can contribute to our understanding of changes in such relationships in complex food chains.

## Figures and Tables

**Table 1:** The size of the five selected passerine prey species, and their proportion among prey items in the diet of the Eurasian Sparrowhawk in the breeding season, and among birds caught at Hel ringing station during spring in 1982–2021. The length and weight of the species after [62,63], proportion in the breeding diet based on literature sources [35,46,53,54,56,57,58,59,60,61], and the proportion among birds ringed at Hel according to the Operation Baltic database (unpublished data). Spring migration timing = spring migration timing of the prey species in northern Europe, after [49].

Species	Length (cm)	Weight (g)	Proportion Among Prey Items	Proportion Among Birds Ringed at Hel	Spring Migration
Song Thrush *Turdus philomelos*	20–22	65–100	3.0–10.5%	6.4%	end of March–mid-May
Eurasian Blackbird *Turdus merula*	23.5–29	80–125	2.0–13.9%	2.9%	mid-March–mid-May
Common Chaffinch *Fringilla coelebs*	14–16	18–29	0.2–22.7%	4.5%	end of March–mid-May
Great Tit *Parus major*	13.5–15	14–22	0.2–17.8%	10.5%	mid-March–mid-May
European Robin *Erithacus rubecula*	12.5–14	14–21	0.2–9.8%	38.3%	end of March–mid-May

**Table 2:** Proportion of prey items of the five passerine species we selected for analyses as the main prey of the Eurasian Sparrowhawk among its prey items during the breeding season, according to the literature sources. “–” = absence of a species in a study, n = the number of prey items collected in a study.

Study Area	Species’ Proportion in the Sparrowhawk Diet						Source (n)
	Song Thrush	Eurasian Blackbird	Common Chaffinch	Great Tit	European Robin	Other Species	
Norway	10.4%	2.5%	10.5%	5.5%	9.8%	61.3%	[59] (n = 2527)
Sweden	10.5%	8.8%	16.8%	1.2%	7.7%	55.0%	[46] (n = 9390)
Finland, Suomenselkä	8.2%	–	22.7%	2.3%	9.3%	57.5%	[58] (n = 772)
Finland, Uusimaa	3.1%	2.0%	12.2%	17.8%	4.8%	60.1%	[60] (n = 902)
Denmark	4.9%	7.7%	7.1%	5.5%	–	74.8%	[35] (n = 34,923)
Germany	2.8%	3.6%	3.4%	5.5%	2.5%	82.2%	[53] (n = 6024)
Poland, Rogów	3.0%	2.8%	4.4%	3.4%	0.3%	86.1%	[56] (n = 930)
Poland, Carpathian Mountains	8.0%	7.2%	6.8%	5.9%	6.0%	66.1%	[61] (n = 1522)
Czech Republic	–	13.9%	6.9%	–	4.4%	74.8%	[54] (n = 115)
Israel	–	11.7%	0.2%	0.2%	0.2%	87.7%	[57] (n = 625)

**Table 3:** Numbers of the Eurasian Sparrowhawk, by age and sex, and of its selected prey species ringed during spring migration (26 March–15 May) of 1982–2021 at Hel ringing station (N Poland). “–” = age categories that were not applied to the five prey species.

Species/Sex and Age Group	N Immatures	N Adults	N Total
Eurasian Sparrowhawk *Accipiter nisus*	1825	519	2344
Females	600	154	754
Males	1225	365	1590
Song Thrush *Turdus philomelos*	–	–	8574
Eurasian Blackbird *Turdus merula*	–	–	3989
Common Chaffinch *Fringilla coelebs*	–	–	5694
Great Tit *Parus major*	–	–	13,564
European Robin *Erithacus rubecula*	–	–	54,916

**Table 4:** Relationships between the timing of the selected quartiles (q25, q50, q75) of spring passage between the Eurasian Sparrowhawk and its prey species, ringed during spring migration (26 March–15 May) at Hel station (N Poland) in 1982–2021. The presented relationships are according to the best multiple regression model. ACNIS = Eurasian Sparrowhawk *Accipiter nisus*, ERRUB = European Robin *Erithacus rubecula*, TUPHI = Song Thrush *Turdus philomelos*. Estimate = coefficient from the multiple regression, SE = standard error of the estimate, W = Wald’s statistics, *p* = statistical significance of the effect, R^2^ = coefficient of determination. Model selection is presented in Table A7, Table A8 and Table A9 in Appendix A.

Species	Estimate	SE	*W*	*p*
ACNIS q25 Best model: F_1,38_ = 11.65, R^2^ = 23.5%				
ERRUB q25	0.52	0.14	14.63	0.0002
Increment	51.73	13.72	14.21	0.0001
ACNIS q50 Best model: F_1,38_ = 4.48, R^2^ = 10.5%				
TUPHI q50	0.36	0.17	4.72	0.0299
Increment	73.94	18.14	16.62	<0.0001
ACNIS q75 Best model: F_1,38_ = 5.13, R^2^ = 11.9%				
ERRUB q75	0.30	0.13	5.40	0.0201
Increment	87.63	14.50	36.53	<0.0001

**Table 5:** Relationships between the dates of the median (q50) for the Eurasian Sparrowhawk spring passage by age and sex and of its prey species, ringed during spring migration (26 March–15 May) at Hel (N Poland) in 1982–2021. The presented relationships are according to the best multiple regression model. ACNIS = Eurasian Sparrowhawk *Accipiter nisus*, ERRUB = European Robin *Erithacus rubecula*, PAMAJ = Great Tit *Parus major*, TUMER = Eurasian Blackbird *Turdus merula*, TUPHI = Song Thrush *Turdus philomelos*. Estimate = coefficients from the multiple regression, SE = standard error of the estimate, W = Wald’s statistics, *p* = statistical significance of the effect. R^2^ = coefficient of determination, AdjR^2^ = adjusted coefficient of determination for the model with more than one explanatory variable. Model selection is presented in Table A10, Table A11, Table A12 and Table A13 in Appendix A.

Species	Estimate	SE	*W*	*p*
ACNIS.MI q50 Best model: F_1,38_ = 6.99, R^2^ = 15.5%				
PAMAJ q50	–0.42	0.155	7.35	0.0067
Increment	158.34	14.17	124.85	<0.0001
ACNIS.MA q50 F_2,33_ = 2.40, AdjR^2^ = 7.4%				
ERRUB q50	0.44	0.19	5.22	0.0223
TUPHI q50	–0.43	0.24	3.28	0.0700
Increment	97.27	16.83	33.42	<0.0001
ACNIS.FI q50 F_1,36_ = 2.85, R^2^ = 7.3%				
TUPHI q50	0.25	0.15	3.01	0.0830
Increment	82.91	15.85	27.36	<0.0001
ACNIS.FA q50 F_1,23_ = 6.80, R^2^ = 22.8%				
TUMER q50	0.68	0.25	7.39	0.0066
Increment	33.64	23.40	2.07	0.1506

**Table 6:** Correlation coefficients (Kendall Tau correlation) between the daily dynamics of the spring migration of the Sparrowhawk (all individuals) and the daily migration dynamics of five prey species in selected springs when more than 100 Sparrowhawks were caught at Hel station (N Poland). Statistically significant correlation coefficients, with significance level *p* < 0.00714 after Bonferroni correction for seven correlations for each species, are marked in bold.

Year/Species	Song Thrush	Eurasian Blackbird	Common Chaffinch	Great Tit	European Robin
1989	**0.60**	–0.04	0.10	–0.01	**0.44**
1996	**0.41**	0.18	**0.28**	0.00	**0.58**
1998	0.11	**–0.32**	**–0.26**	**–0.31**	0.18
1999	0.26	–0.01	0.13	–0.01	0.22
2000	**0.36**	**–0.30**	0.17	–0.23	**0.29**
2002	**0.37**	–0.17	–0.04	**–0.43**	0.17
2003	0.24	0.04	0.11	0.02	0.26

**Table 7:** Relationships between the dates of the median (q50) for the Eurasian Sparrowhawk spring passage, by sex and age, caught during spring migration (26 March–15 May) at Hel (N Poland) in 1982–2021, and temperatures in February–April in regions K1 and K2 (Figure 1). The presented relationships are according to the best multiple regression model for the median date of passage (q50) for each sex/age group of Sparrowhawks: ACNIS.MI = immature males, ACNIS.MA = adult males, ACNIS.FI = immature females, ACNIS.FA = adult females. Estimate = coefficients from multiple regression, SE = standard error of the estimate, W = Wald’s statistics, *p* = statistical significance of the effect, R^2^ = coefficient of determination. Model selection is presented in Table A14, Table A15, Table A16 and Table A17 in Appendix A.

Sex/Age Group	Estimate	SE	*W*	*p*
ACNIS.MI q50 Best model: F_1,38_ = 4.95, R^2^ = 11.5%				
K2 April	–1.27	0.56	5.21	0.0225
Increment	127.99	3.58	1277.48	<0.0001
ACNIS.MA q50 F_1,34_ = 3.90, R^2^ = 10.3%				
K1 March	–1.02	0.50	4.13	0.0421
Increment	104.53	3.40	947.40	<0.0001
ACNIS.FI q50 F_1,36_ = 1.56, R^2^ = 4.1%				
K1 March	0.61	0.48	1.64	0.2000
Increment	106.28	3.26	1062.50	<0.0001
ACNIS.FA q50 F_1,23_ = 3.28, R^2^ = 12.5%				
K1 February	–1.03	0.55	3.57	0.0588
Increment	101.61	2.58	1554.36	0.0000

**Figure A1:**
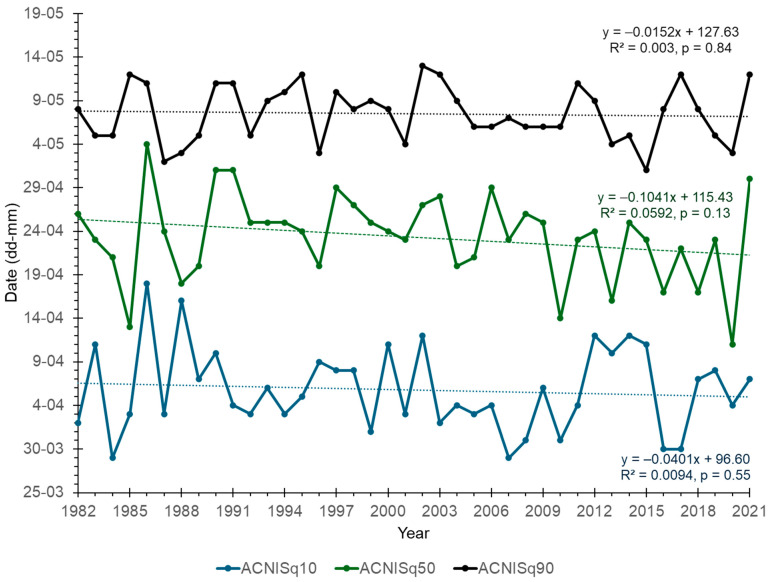
Multi-year trends in the dates of the beginning (q10), median (q50) and end (q90) of spring passage of Sparrowhawks (all individuals jointly) caught during spring migration (26 March–15 May) at the Hel ringing station in 1982–2021. None of the trend regression equations (dotted lines) was significant; the details of the regression are presented in colour corresponding to the relevant regression line.

## Data Availability

Data contained in the article are available in the public domain resources. Data from ringing at Operation Baltic ringing stations can be found at the Global Biodiversity Information Facility (GBIF) database at Ringing Data from the Bird Migration Research Station, University of Gdańsk (Occurrence dataset https://doi.org/10.15468/q5o88l) [87] accessed via GBIF.org on 8 July 2025.

## Appendix A

**Table A1:** Pearson’s correlation coefficients of the temperatures in February–April in 1982–2021 in squares K1 and K2 (Figure 1), which we used as explanatory variables in multiple regression models. Significant correlations after Bonferroni correction for multiple comparisons (*p* < 0.00714) are marked in **bold** font.

	K1_MAR	K2_MAR	K2_APR
**K1_FEB**	0.43	**0.56**	0.34
**K1_MAR**		**0.80**	0.34
**K2_MAR**			0.37

**Table A2:** Number of Sparrowhawks and selected prey species caught in consecutive spring seasons (26 March–15 May) in 1982–2021 at the Hel bird ringing station (N Poland). Years marked in **bold**—years in which more than 100 Sparrowhawks were caught, selected to determine the correlations between their daily migration dynamics and that of the Sparrowhawk (treated jointly). Years marked in grey were excluded from analyses, because fewer than 10 individuals were caught.

Year/ Species	Eurasian Sparrowhawk	Song Thrush	Eurasian Blackbird	Common Chaffinch	European Robin	Great Tit
1982	10	292	120	209	1219	775
1983	39	227	99	223	484	200
1984	21	178	152	141	653	267
1985	14	105	38	63	525	148
1986	44	187	27	94	511	171
1987	35	69	37	94	450	192
1988	16	75	7	32	205	93
**1989**	139	137	12	56	282	24
1990	57	148	4	82	254	8
1991	91	102	12	51	194	44
1992	65	62	5	54	103	21
1993	93	111	10	76	405	33
1994	49	92	9	66	257	61
1995	21	121	44	158	1225	539
**1996**	113	216	341	177	3623	391
1997	94	150	76	215	1362	484
**1998**	162	196	202	84	1513	162
**1999**	116	170	82	132	1590	49
**2000**	105	199	138	164	1114	217
2001	94	157	65	162	590	115
**2002**	112	231	116	217	1737	169
**2003**	106	309	128	232	2326	81
2004	66	239	88	115	2377	535
2005	18	208	122	167	1543	1207
2006	62	217	290	133	1109	731
2007	39	366	86	184	1990	144
2008	64	234	105	112	1789	1464
2009	55	336	138	153	2786	522
2010	50	141	61	126	1100	56
2011	54	261	121	141	1766	689
2012	58	244	32	253	1890	36
2013	25	150	288	145	910	1767
2014	33	233	23	102	1335	70
2015	46	219	104	145	1074	259
2016	31	337	167	180	2360	1097
2017	41	230	86	118	1980	53
2018	33	305	193	181	1367	177
2019	23	609	118	248	4673	108
2020	15	345	112	208	2414	73
2021	35	366	131	201	1831	332

**Table A3:** Bird pluckings collected near Hel bird ringing station during 26 March–12 May 2024. The results are summarised in Figure 3A.

No	Date	Species	Scientific name
1	28 March 2024	Great Spotted Woodpecker	*Dendrocopos major*
2	29 March 2024	Eurasian Blackbird	*Turdus merula*
3	29 March 2024	Song Thrush	*Turdus philomelos*
4	30 March 2024	Great Tit	*Parus major*
5	30 March 2024	Redwing	*Turdus iliacus*
6	30 March 2024	Eurasian Blackbird	*Turdus merula*
7	31 March 2024	Song Thrush	*Turdus philomelos*
8	31 March 2024	Eurasian Blackbird	*Turdus merula*
9	31 March 2024	Great Tit	*Parus major*
10	31 March 2024	Great Tit	*Parus major*
11	1 April 2024	Eurasian Blackbird	*Turdus merula*
12	1 April 2024	European Robin	*Erithacus rubecula*
13	1 April 2024	European Robin	*Erithacus rubecula*
14	1 April 2024	Great Tit	*Parus major*
15	1 April 2024	Eurasian Blackbird	*Turdus merula*
16	1 April 2024	Great Tit	*Parus major*
17	1 April 2024	Song Thrush	*Turdus philomelos*
18	1 April 2024	Redwing	*Turdus iliacus*
19	1 April 2024	European Robin	*Erithacus rubecula*
20	1 April 2024	Great Tit	*Parus major*
21	1 April 2024	Great Tit	*Parus major*
22	2 April 2024	Eurasian Blackbird	*Turdus merula*
23	6 April 2024	Eurasian Blackbird	*Turdus merula*
24	20 April 2024	Eurasian Blue Tit	*Cyanistes caeruleus*
25	20 April 2024	Eurasian Blue Tit	*Cyanistes caeruleus*
26	20 April 2024	Great Tit	*Parus major*
27	20 April 20244	Song Thrush	*Turdus philomelos*
28	20 April 2024	Great Tit	*Parus major*
29	20 April 2024	Great Tit	*Parus major*
30	20 April 2024	Eurasian Blackbird	*Turdus merula*
31	20 April 2024	Song Thrush	*Turdus philomelos*
32	25 April 2024	Eurasian Blackbird	*Turdus merula*
33	26 April 2024	Common Chaffinch	*Fringilla coelebs*
34	1 May 2024	Song Thrush	*Turdus philomelos*
35	1 May 2024	Song Thrush	*Turdus philomelos*
36	1 May 2024	Eurasian Blackbird	*Turdus merula*
37	2 May 2024	Lesser Spotted Woodpecker	*Dryobates minor*
38	2 May 2024	Eurasian Skylark	*Alauda arvensis*
39	3 May 2024	Great Tit	*Parus major*
40	3 May 2024	Great Tit	*Parus major*
41	3 April 2024	Eurasian Blue Tit	*Cyanistes caeruleus*
42	7 May 2024	Eurasian Skylark	*Alauda arvensis*
43	7 May 2024	Great Tit	*Parus major*
44	7 May 2024	Song Thrush	*Turdus philomelos*
45	7 May 2024	Great Tit	*Parus major*
46	7 May 2024	Great Spotted Woodpecker	*Dendrocopos major*
47	7 May 2024	Hawfinch	*Coccothraustes coccothraustes*
48	12 May 2024	Eurasian Blackbird	*Turdus merula*

**Table A4:** Sparrowhawks’ prey during attacks observed near the Hel bird ringing station during 26 March–12 May 2024. F = female, M = male, I = immature, A = adult, “–” = a lack of information about Sparrowhawk’s sex or age. The results are summarised in Figure 3B.

No	Date	Species	Scientific Name	Sex and Age of Sparrowhawk	Type of Observation
1	26 March 2024	*Regulus* sp.	*Regulus* sp.	M	Observation of chase after prey
2	27 March 2024	Eurasian Blackbird	*Turdus merula*	F, I	Mist net
3	27 March 2024	European Robin	*Erithacus rubecula*	–	Mist net
4	27 March 2024	Great Tit	*Parus major*	M	Mist net
5	30 March 2024	Northern Wren	*Troglodytes troglodytes*	M, A	Mist net
6	5 April 2024	Song Thrush	*Turdus philomelos*	–	Mist net
7	7 April 2024	Song Thrush	*Turdus philomelos*	–	Mist net
8	8 April 2024	Song Thrush	*Turdus philomelos*	F, A	Mist net
9	11 April 2024	European Robin	*Erithacus rubecula*	–	Sparrowhawk attack
10	14 April 2024	European Robin	*Erithacus rubecula*	–	Mist net
11	20 April 2024	Common Chaffinch	*Fringilla coelebs*	–	Mist net
12	21 April 2024	European Robin	*Erithacus rubecula*	–	Mist net
13	21 April 2024	Redwing	*Turdus iliacus*	I	Mist net
14	26 April 2024	Eurasian Blackcap	*Sylvia atricapilla*	A	Mist net
15	28 April 2024	Song Thrush	*Turdus philomelos*	–	Mist net
16	28 April 2024	Eurasian Blackbird	*Turdus merula*	–	Mist net

**Table A5:** Summary statistics for linear regressions of the median (q50) dates of the Eurasian Sparrowhawk spring migration in 1982–2021 at the Hel ringing station (N Poland). The linear regressions are presented in Figure 6. *β* Slope = regression coefficient, SE = standard error; R^2^—determination coefficient, t, *p* = results of *t*-test, *p* < 0.05 marked in bold face, 40 × *β* = estimated change in days of migration timing over 1982–2021, negative values reflect shift to earlier migration.

Sex/Age Group	*β* Slope	SE	R^2^	t	*p*	40 Years × *β* (Days)
ACNIS.MI	−0.11	0.06	**0.09**	−1.94	0.06	−4.4
ACNIS.MA	−0.07	0.07	0.03	−1.01	0.32	−2.8
ACNIS.FI	−0.05	0.06	0.02	−0.82	0.42	−2.0
ACNIS.FA	−0.13	0.12	0.05	−1.06	0.30	−5.2

**Table A6:** Summary statistics for linear regressions of the median (q50) dates of the prey species spring migration in 1982–2021 at the Hel ringing station (N Poland). *β* Slope = regression coefficient, SE = standard error; R^2^ = determination coefficient, t, *p* = results of *t*-test, *p* < 0.05 marked in bold face, 40 × *β* = estimated change in days of migration timing over 1982–2021, negative values reflect shift to earlier migration.

Species	*β* Slope	SE	R^2^	t	*p*	40 Years × *β* (Days)
Song Thrush	−0.02	0.06	**0.00**	−0.32	0.75	−0.8
Eurasian Blackbird	−0.05	0.06	0.01	−0.75	0.46	−2.0
Common Chaffinch	−0.05	0.08	0.01	−0.59	0.56	−2.0
Great Tit	0.07	0.06	0.04	1.22	0.23	2.8
European Robin	−0.10	0.08	0.05	−1.34	0.19	−4.0

**Table A7:** Model selection procedure by “all subsets”, according to AIC, from the full model of the relationship between the dates of the first quartile (q25) of spring migration of the Eurasian Sparrowhawk and its prey species, ringed during spring migration (26 March–15 May) in 1982–2021 at the Hel ringing station (N Poland). ACNIS = Eurasian Sparrowhawk *Accipiter nisus*, FRCOE = Chaffinch *Fringilla coelebs,* PAMAJ = Great Tit *Parus major*, TUMER = Blackbird *Turdus merula*, TUPHI = Song Thrush *Turdus philomelos*. The table presents all models with ΔAIC < 2. The models ranked by Akaike’s Information Criteria (AIC), k is the number of estimated parameters in the model, ∆AIC gives the difference in AICc from the model with the lowest AIC. The best model, presented in Table 4 and discussed in the text, is marked in **bold** face.

Model Formula	*k*	AIC	ΔAIC
ACNIS ~ ERRUB	1	243.40	0.00
ACNIS ~ ERRUB + TUMER	2	244.44	1.04
ACNIS ~ ERRUB + PAMAJ	2	245.38	1.98
ACNIS ~ ERRUB + FRCOE	2	245.39	1.99
ACNIS ~ ERRUB + TUPHI	2	245.40	2.00

**Table A8:** Model selection procedure by “all subsets”, according to AIC, from the full model of the relationship between the dates of the median (q50) of spring migration of the Eurasian Sparrowhawk and its prey species, ringed during spring migration (26 March–15 May) in 1982–2021 at the Hel ringing station (N Poland). Symbols as in Table A7.

Model Formula	*k*	AIC	ΔAIC
ACNIS ~ TUPHI	1	242.86	0.00
ACNIS ~ TUPHI + PAMAJ	2	242.88	0.03
ACNIS ~ FRCOE + TUPHI + PAMAJ	3	244.08	1.22
ACNIS ~ TUMER + TUPHI + PAMAJ	3	244.18	1.32
ACNIS ~ FRCOE + TUPHI	2	244.41	1.55
ACNIS ~ ERRUB + TUPHI	2	244.85	1.97

**Table A9:** Model selection procedure by “all subsets”, according to AIC, from the full model of the relationship between the dates of the third quartile (q75) of spring migration of the Eurasian Sparrowhawk and its prey species, ringed during spring migration (26 March–15 May) in 1982–2021 at the Hel ringing station (N Poland). Symbols as in Table A7.

Model Formula	*k*	AIC	ΔAIC
ACNIS ~ ERRUB	1	225.00	0.00
ACNIS ~ ERRUB + PAMAJ	2	226.74	1.74
ACNIS ~ ERRUB + FRCOE	2	226.78	1.78
ACNIS ~ ERRUB + TUMER	2	226.98	1.98
ACNIS ~ ERRUB + TUPHI	2	227.00	2.00

**Table A10:** Model selection procedure by “all subsets”, according to AIC, from the full model of the relationship between the dates of the median (q50) of spring migration of the immature males of the Eurasian Sparrowhawk and its prey species, ringed during spring migration (26 March–15 May) in 1982–2021 at the Hel ringing station (N Poland). Symbols as in Table A7.

Model Formula	*k*	AIC	ΔAIC
ACNIS.MI ~ PAMAJ	1	229.04	0.00
ACNIS.MI ~ TUMER + PAMAJ	2	230.74	1.70
ACNIS.MI ~ FRCOE + PAMAJ	2	230.80	1.76
ACNIS.MI ~ ERRUB + PAMAJ	2	230.90	1.86
ACNIS.MI ~ TUPHI + PAMAJ	2	230.91	1.87

**Table A11:** Model selection procedure by “all subsets”, according to AIC, from the full model of the relationship between the dates of the median (q50) of spring migration of the adult males of the Eurasian Sparrowhawk and its prey species, ringed during spring migration (26 March–15 May) in 1982–2021 at the Hel ringing station (N Poland). Symbols as in Table A7.

Model Formula	*K*	AIC	ΔAIC
ACNIS.MA ~ ERRUB + TUPHI	2	213.92	0.00
ACNIS.MA ~ ERRUB + TUPHI + PAMAJ	3	215.04	1.12
ACNIS.MA ~ ERRUB	1	215.06	1.14
ACNIS.MA ~ PAMAJ	1	215.56	1.64
ACNIS.MA ~ ERRUB + FRICOE + TUPHI	3	215.63	1.71
ACNIS.MA ~ ERRUB + TUMER + TUPHI	3	215.87	1.95

**Table A12:** Model selection procedure by “all subsets”, according to AIC, from the full model of the relationship between the dates of the median (q50) of spring migration of the immature females of the Eurasian Sparrowhawk and its prey species, ringed during spring migration (26 March–15 May) in 1982–2021 at the Hel ringing station (N Poland). Symbols as in Table A7.

Model Formula	*K*	AIC	ΔAIC
ACNIS.FI ~ TUPHI	1	219.36	0.00
ACNIS.FI ~ ERRUB	1	220.28	0.92
ACNIS.FI ~ TUMER + TUPHI	2	221.29	1.93
ACNIS.FI ~ ERRUB + TUPHI	2	221.31	1.95
ACNIS.FI ~ FRICOE + TUPHI	2	221.33	1.97
ACNIS.FI ~ TUPHI + PAMAJ	2	221.34	1.98

**Table A13:** Model selection procedure by “all subsets”, according to AIC, from the full model of the relationship between the dates of the median (q50) of spring migration of the adult females of the Eurasian Sparrowhawk and its prey species, ringed during spring migration (26 March–15 May) in 1982–2021 at the Hel ringing station (N Poland). Symbols as in Table A7.

Model Formula	*K*	AIC	ΔAIC
ACNIS.FA ~ TUMER	1	158.69	0.00
ACNIS.FA ~ TUMER + TUPHI	2	158.85	0.16
ACNIS.FA ~ TUMER + PAMAJ	2	159.31	0.62
ACNIS.FA ~ FRCOE + TUMER	2	159.61	0.92
ACNIS.FA ~ FRCOE + TUMER + PAMAJ	3	159.83	1.14
ACNIS.FA ~ TUMER + TUPHI + PAMAJ	3	159.90	1.21
ACNIS.FA ~ ERRUB + TUMER + TUPHI	3	159.95	1.26
ACNIS.FA ~ PAMAJ	1	160.20	1.51
ACNIS.FA ~ FRCOE + TUMER + TUPHI	3	160.26	1.57
ACNIS.FA ~ ERRUB + TUMER	2	160.51	1.82

**Table A14:** Model selection procedure by “all subsets”, according to AIC, from the full model of the relationship between the dates of the median (q50) of spring migration of immature males Eurasian Sparrowhawk, ringed during spring migration (26 March–15 May) in 1982–2021 at the Hel ringing station (N Poland). and temperatures in February–April in regions K1 and K2 (Figure 1). The table presents all models with ΔAIC < 2. The models ranked by Akaike’s Information Criteria (AIC), k is the number of estimated parameters in the model, ∆AIC gives the difference in AICc from the model with the lowest AIC. The best model, presented in Table 7 and discussed in the text, is marked in bold face.

Model Formula	*k*	AIC	ΔAIC
ACNIS.MI ~ K2 April	1	230.89	0.00
ACNIS.MI ~ K2 April + K1 February	2	231.84	0.95
ACNIS.MI ~ K2 April + K1 March	2	232.78	1.22

**Table A15:** Model selection procedure by “all subsets”, according to AIC, from the full model of the relationship between the dates of the median (q50) of spring migration of adult males Eurasian Sparrowhawk, ringed during spring migration (26 March–15 May) in 1982–2021 at the Hel ringing station (N Poland) and temperatures in February–April in regions K1 and K2 (Figure 1). Symbols as in Table A14.

Model Formula	*k*	AIC	ΔAIC
ACNIS.MA ~ K1 March	1	212.90	0.00
ACNIS.MA ~ K1 February + K1 March	2	213.97	1.07
ACNIS.MA ~ K1 February	1	214.14	1.24
ACNIS.MA ~ K2 April + K1 March	2	214.88	1.98

**Table A16:** Model selection procedure by “all subsets”, according to AIC, from the full model of the relationship between the dates of the median (q50) of spring migration of immature females Eurasian Sparrowhawk, ringed during spring migration (26 March–15 May) in 1982–2021 at the Hel ringing station (N Poland) and temperatures in February–April in regions K1 and K2 (Figure 1). Symbols as in Table A14.

Model Formula	*k*	AIC	ΔAIC
ACNIS.FI ~ K1 March	1	220.64	0.00
ACNIS.FI ~ K1 February + K1 March	2	221.03	0.39
ACNIS.FI ~ K2 April + K1 March	2	221.62	0.98
ACNIS.FI ~ K1 February	1	221.89	1.25
ACNIS.FI ~ K2 April	1	221.96	1.32
ACNIS.FI ~ K2 April + K1 March	3	222.48	1.83

**Table A17:** Model selection procedure by “all subsets”, according to AIC, from the full model of the relationship between the dates of the median (q50) of spring migration of adult females Eurasian Sparrowhawk ringed during spring migration (26 March–15 May) in 1982–2021 at the Hel ringing station (N Poland) and temperatures in February–April in regions K1 and K2 (Figure 1). Symbols as in Table A14.

Model Formula	*k*	AIC	ΔAIC
ACNIS.FA ~ K1 February	1	161.83	0.00
ACNIS.FA ~ K4 April + K1 February	2	163.06	1.23
ACNIS.FA ~ K1 February + K1 March	2	163.82	2.00
